# Сложность дифференциального диагноза объемных образований надпочечника при врожденной дисфункции коры надпочечников: серия клинических случаев

**DOI:** 10.14341/probl13564

**Published:** 2025-05-20

**Authors:** А. Шевэ, Н. В. Тарбаева, Ю. Ю. Голубкина, М. М. Гаджимурадова, К. В. Иващенко, Д. О. Ладыгина, М. В. Воронцова, О. Б. Безлепкина, Г. А. Мельниченко, Н. Г. Мокрышева

**Affiliations:** Национальный медицинский исследовательский центр эндокринологии; Национальный медицинский исследовательский центр эндокринологии; Национальный медицинский исследовательский центр эндокринологии; Национальный медицинский исследовательский центр эндокринологии; Национальный медицинский исследовательский центр эндокринологии; Первый Московский государственный медицинский университет им. И.М. Сеченова (Сеченовский Университет); Национальный медицинский исследовательский центр эндокринологии; Московский государственный университет им. М.В. Ломоносова; Национальный медицинский исследовательский центр эндокринологии; Национальный медицинский исследовательский центр эндокринологии; Национальный медицинский исследовательский центр эндокринологии

**Keywords:** врожденная дисфункция коры надпочечников, TART, заместительная терапия, надпочечниковая недостаточность, надпочечники

## Abstract

Врожденная дисфункция коры надпочечников (ВДКН) — это группа аутосомно-рецессивных заболеваний, характеризующихся дефектом одного из ферментов, принимающих участие в стероидогенезе, наиболее распространенным из которых является дефицит 21-гидроксилазы вследствие мутаций в гене CYP21A2. С момента внедрения заместительной терапии и программ неонатального скрининга в 1950-х годах наблюдается значительное улучшение показателей выживаемости среди новорожденных, страдающих ВДКН. Однако, несмотря на достигнутый прогресс, уровень смертности, связанный с данным заболеванием, продолжает оставаться на относительно высоком уровне. Терапевтическое лечение, направленное на достижение медикаментозной компенсации, представляет собой сложную задачу, что связано с различными долгосрочными осложнениями, возникающими как в результате самого заболевания, так и его лечения. Эти осложнения затрагивают целый ряд физиологических функций, включая метаболизм, рост и развитие, а также состояние сердечно-сосудистой системы и фертильность, что подчеркивает необходимость дальнейших исследований и совершенствования подходов к лечению названной патологии.

В данной статье представлена серия из 4 клинических случаев пациентов с ВДКН, длительно находившихся в состоянии декомпенсации глюко- и минералокортикоидной недостаточности, у которых наблюдалось развитие объемных образований надпочечников значительных размеров, а также TART опухолей яичек.

## Abstract (en)

Congenital adrenal hyperplasia (CAH) encompasses a group of autosomal recessive disorders characterized by defects in enzymes critical for steroidogenesis, with 21-hydroxylase deficiency due to mutations in the CYP21A2 gene being the most prevalent form.

Since the introduction of replacement therapy and neonatal screening programs in the 1950s, there has been a significant increase in survival rates among newborns diagnosed with CAH. However, despite these advancements, mortality associated with this condition remains disproportionately high. Achieving optimal therapeutic compensation through medication remains a complex challenge, contributing to a range of long-term complications. These complications stem from both the underlying disease and its treatment, impacting key physiological functions, including metabolism, growth and development, cardiovascular health, and fertility. These multifaceted outcomes underscore the need for ongoing research and the refinement of therapeutic approaches to better manage this intricate condition. This article presents a series of four clinical cases of CAH characterized by the absence of sustained compensation for glucoand mineralocorticoid deficiencies. These cases were further complicated by the development of large adrenal masses and ectopic testicular adrenal rest tissue (TART), emphasizing the challenges in achieving long-term disease management.

## ВВЕДЕНИЕ

Врожденная дисфункция коры надпочечников (ВДКН) — это группа аутосомно-рецессивных заболеваний, которая характеризуется дефектом одного из ферментов стероидогенеза. Наиболее распространенным является дефицит 21-гидроксилазы вследствие мутаций в гене CYP21A2. Фенотипически заболевание классифицируется на 3 формы в зависимости от выраженности клинических проявлений: классическую (сольтеряющую и вирильную) и неклассическую формы, и коррелирует со степенью снижения активности 21-гидроксилазы при различных мутациях в гене CYP21A2. Мутации, полностью инактивирующие этот фермент, ассоциированы с развитием сольтеряющей формы, при снижении активности фермента до ~2% развивается простая вирильная форма, в то время как в случае сохранения активности в пределах 20–70% — мягкая неклассическая форма ВДКН [1].

Общая распространенность классических форм ВДКН в среднем составляет от 1:14000 до 1:19500 новорожденных. При этом для заболевания характерна этническая вариативность с наибольшей встречаемостью в небольших, генетически изолированных группах [2]. По данным неонатального скрининга, в Российской Федерации частота классических форм дефицита 21-гидроксилазы составляет 1 случай на 9 638 живых новорожденных, что чаще, чем в среднем в мировой популяции, и характеризуется равным распределением среди мальчиков и девочек [3].

Основным биомаркером для диагностики ВДКН, обусловленной дефицитом 21-гидроксилазы, является 17α-гидроксипрогестерон (17OHP), который является субстратом для 21-гидроксилазы и, таким образом, прямым предшественником стероидов, накапливающимся до ферментативного блока. При классических формах уровень 17OHP превышает 300 нмоль/л (100 нг/мл). Для окончательного установления диагноза проводится генетическое исследование, направленное на выявление мутаций в гене CYP21A2. В отношении неклассических форм, при получении базальных значений 17ОНР, превышающих 30 нмоль/л (10 нг/мл), диагноз считается подтвержденным. В случае пограничных значений 17ОНР (6–30 нмоль/л (2–10 нг/мл)), необходимо проведение дополнительного стимулирующего теста с синактеном (косинтропином, тетракозактидом — синтетическим аналогом адренокортикотропного гормона, АКТГ), который считается золотым стандартом диагностики нВДКН и признается общепринятым в мировой практике [4].

Лечение классических форм ВДКН представляет собой сложную задачу и требует пожизненного применения заместительной терапии. Основные компоненты лечения включают глюкокортикоиды (ГКС), такие как гидрокортизон или преднизолон, которые используются для замещения дефицита кортизола. При наличии сольтеряющей формы заболевания также применяются минералокортикостероиды (МКС), например, флудрокортизон, для обеспечения адекватного контроля за уровнями электролитов и артериальным давлением. Терапия ГКС является основой лечения ВДКН и преследует две цели: восполнить дефицит кортизола и препятствовать гиперсекреции АКТГ, которая приводит к избыточной продукции андрогенов [5].

Сложность терапии заключается в том, что на сегодняшний день не разработаны препараты или терапевтические протоколы, которые бы позволили точно воспроизвести физиологический суточный ритм секреции кортизола. Выбор оптимальных доз ГКС и МКС представляет собой трудную задачу. Осложнения, связанные с классической формой ВДКН, можно условно разделить на две группы: осложнения, обусловленные передозировкой и побочными эффектами ГКС (увеличение риска сердечно-сосудистых заболеваний, ожирения и патологии костной ткани), и состояния, возникающие вследствие недостаточности дозы ГКС [6]. Для оценки степени компенсации при сольтеряющей форме ВДКН рекомендуют использовать следующие ключевые показатели: 17ОНР, тестостерон, андростендион, гонадотропины — для оценки адекватности замещения глюкокортикоидной недостаточности, а ренин и электролиты (K, Na) — для оценки компенсации минералокортикоидной недостаточности [4].

Отсутствие адекватной компенсации глюкокортикоидной и минералокортикоидной недостаточности у пациентов с ВДКН может привести к серьезным последствиям, включая развитие надпочечниковой недостаточности, рост различных образований надпочечников и тестикулярных аденом (TART). Нарушение синтеза кортизола приводит к снижению его воздействия на аденогипофиз по отрицательной обратной связи и, следовательно, увеличению секреции АКТГ, стимуляции коры надпочечников с последующим накоплением продуктов стероидогенеза выше ферментативного блока: 17OHP и надпочечниковых андрогенов — андростендиона и тестостерона [7].

В данной серии клинических наблюдений представлены пациенты с ВДКН, у которых на фоне длительной неудовлетворительной компенсации заболевания развились осложнения.

## Клинический случай №1

Пациент Д., 33 года (19.08.1988 г.), в 2022 г. обратился в ФГБУ «НМИЦ эндокринологии» Минздрава России для обследования и лечения с жалобами на общую слабость, боль в поясничной области преимущественно слева, невозможность согнуться, ощущение дискомфорта («поддавливания») в левом подреберье, усиливающиеся после приема пищи.

С рождения наблюдался по поводу сольтеряющей формы ВДКН. Диагноз установлен клинически, подтвержден результатами молекулярно-генетического анализа: обнаружена генная конверсия экзонов 1–3 в гомозиготном состоянии в гене CYP21A2. Родственники здоровы. Находился на постоянной терапии ГКС и МКС, с 13 лет прием препаратов самостоятельно отменил. Со слов больного, на фоне отмены состояние удовлетворительное, кризов надпочечниковой недостаточности не отмечал. В 2021 г. в связи с болевым синдромом в спине обратился в лечебной учреждение по месту жительства. Проведена мультиспиральная компьютерная томография (МСКТ) органов брюшной полости с внутривенным контрастированием, по данным которой надпочечники увеличены в размерах за счет многоузловых жиросодержащих образований в левом надпочечнике размерами 23х13,9х16,4 см и в правом надпочечнике размером 8,8х6,7х11,9 см. По данным гормонального обследования: 17ОНП — 1397 нмоль/л, ренин — более 500 мЕд/л, АКТГ — 570 пг/мл. Консультирован эндокринологом, рекомендована заместительная терапия, госпитализация в специализированный стационар. От приема ГКС, МКС отказывался, мотивируя удовлетворительным самочувствием. На момент госпитализации в ФГБУ «НМИЦ эндокринологии» заместительной терапии не получал.

## Данные физикального обследования

Общее состояние удовлетворительное. Масса тела — 86,5 кг. Рост — 160 см. Индекс массы тела (ИМТ) — 33,8 кг/м² (ожирение I). ЧСС — 93 уд./мин. Артериальное давление — 130/90 мм рт.ст. При пальпации живота, в проекции левого латерального канала, уходящее в левое подреберье — объемное образование не менее 20 см, плотной консистенции, безболезненное при пальпации. В мошонке пальпаторно определяются образования яичек «каменистой» плотности 4–5 см. По остальным органам и системам без особенностей.

Данные гормонального обследования, электролитов представлены в таблице 1.

**Table:** Таблица 1. Данные лабораторного обследования пациента №1

Показатель	Результат	Референсный интервал	Единицы измерения
Биохимический анализ крови
Натрий	139	136–145	ммоль/л
Хлориды	100,3	98–107	ммоль/л
Калий	5,15>	3,5–5,1	ммоль/л
Глюкоза	5,06	3,1–6,1	ммоль/л
Гормональный анализ крови
Тестостерон	31,2>	11–28,2	нмоль/л
ФСГ	5,2	1,6–9,7	Ед/л
ЛГ	0,216<	2,5–11	Ед/л
СССГ	33,1	18,3–54,1	нмоль/л
Ренин (прямой)	500,0>	2,8–39,9	мЕд/л
Кортизол (утро)	340,8	171–536	нмоль/л
17-OH прогестерон	148,7>	0,6–11,8	нмоль/л
АКТГ (утро)	454,8>	7,2–63,3	пг/мл

Результаты других лабораторных исследований: общеклинического и биохимического анализов крови (АЛТ, АСТ, креатинин, общий и прямом билирубин, липидный спектр, общий белок, альбумин), коагулограммы и общего анализа мочи — без особенностей.

В ходе госпитализации в терапевтическом отделении с учетом наличия по данным анамнеза образований надпочечников и яичек проведено контрольное динамическое обследование.

## Данные инструментального обследования

По данным МСКТ надпочечников с внутривенным контрастированием от 02.03.2022 г., левый надпочечник замещен крупным многоузловым жиросодержащим образованием размерами 26х14х17 см с наличием в структуре кальцинатов размером до 8 мм, оттесняющим вправо петли тонкой кишки, двенадцатиперстную кишку, поджелудочную железу, брыжейку тонкой кишки, верхнюю брыжеечную артерию, чревный ствол, влево — поперечный отдел ободочной кишки, селезенку, смещающим почку книзу с ротацией ворот, и с плотностью перегородок и солидного компонента по фазам: 30-45-165-60 HU (нативная — артериальная — венозная — отсроченная фазы соответственно), абсолютный коэффициент вымывания — 78%, относительный коэффициент вымывания — 63%; правый надпочечник замещен многоузловым жиросодержащим образованием размерами 7,5х4,2х6,5 см в медиальной ножке, 7,7х3,5х8,7 см в латеральной ножке, 3,5 см в теле, прилежащем к правой доле печени, плотностью по фазам сканирования 26-40-65-60 HU (нативная — артериальная — венозная — отсроченная фазы соответственно) (рис. 1).

**Figure:**
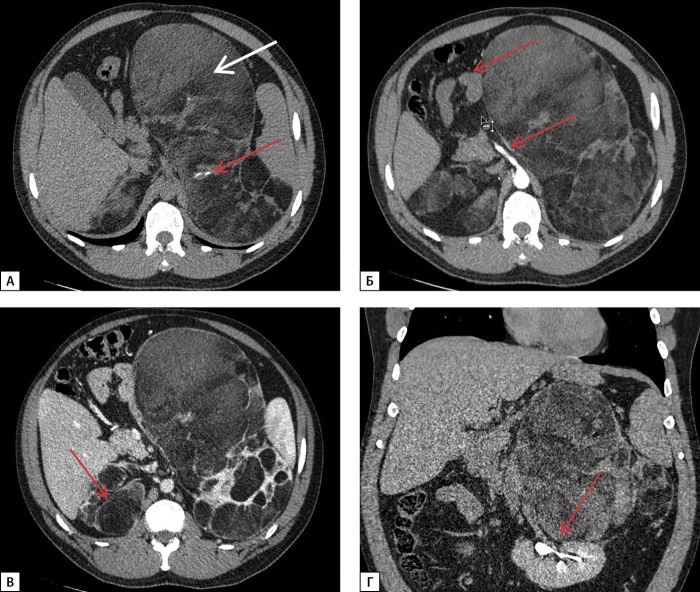
Рисунок 1. МСКТ надпочечников с контрастным усилением: А — нативная фаза — левый надпочечник замещен крупным многоузловым жиросодержащим образованием (белая стрелка), с перегородками, в структуре образования кальцинаты (красная стрелка); Б — артериальная фаза — образование левого надпочечника оттесняет вправо петли тонкой кишки, двенадцатиперстную кишку, поджелудочную железу, брыжейку тонкой кишки, верхнюю брыжеечную артерию, чревный ствол (красные стрелки); В — венозная фаза — правый надпочечник замещен многоузловым жиросодержащим образованием с перегородками, прилежащим к правой доле печени (красная стрелка); Г — отсроченная фаза — образование левого надпочечника смещает почку книзу с ротацией ворот, оттесняет поперечный отдел ободочной кишки, селезенку влево (красная стрелка).

При УЗИ мошонки от 04.03.2022 г. в правом яичке визуализировались множественные образования размером до 25 мм, в левом яичке — множественные образования размером до 20 мм, свободная жидкость в оболочках не лоцировалась. С учетом анамнеза, образования расценены как двусторонние TART-опухоли.

В стационаре инициирована заместительная терапия гидрокортизоном в суточной дозе 15 мг. Учитывая наличие синдрома механической компрессии органов брюшной полости, консультирован эндокринным хирургом, принято решение о проведении левосторонней адреналэктомии открытым доступом.

18.04.2022 г. проведена левосторонняя открытая адреналэктомия. При ревизии в проекции селезеночного угла определялась опухоль левого надпочечника до 25 см в диаметре, вишневого цвета в капсуле, подрастающей к петлям тонкой кишки, телу и сосудистому пучку селезенки, верхнему полюсу левой почки, диафрагме. Тупым и острым путем с использованием Harmonic Focus левый надпочечник с опухолью выделен из ложа и отсечен.

Послеоперационный период протекал без осложнений. Пациент выписан в удовлетворительном состоянии.

При макроскопическом исследовании послеоперационного материала описан левый надпочечник размерами 23,0х19,0х8,0 см, с гладкой, желтоватой (жирового вида) поверхностью с бурыми участками. На разрезе слои коры надпочечника визуально не определялись, ткань пестрого вида с участками желтоватого и бурого цветов 1,5–8,0 см в наибольшем измерении (рис. 2). По данным микроскопического исследования, надпочечник представлен корковым веществом с расширенной пучковой зоной, содержащей небольшое количество онкоцитарных клеток, разделенных толстыми соединительнотканными прослойками, в строме описаны очаги лимфоидной инфильтрации, хромаффинные клетки не определялись. Морфологическая картина соответствовала диффузной гиперплазии надпочечника с обширными участками миелолипоматозной метаплазии.

**Figure:**
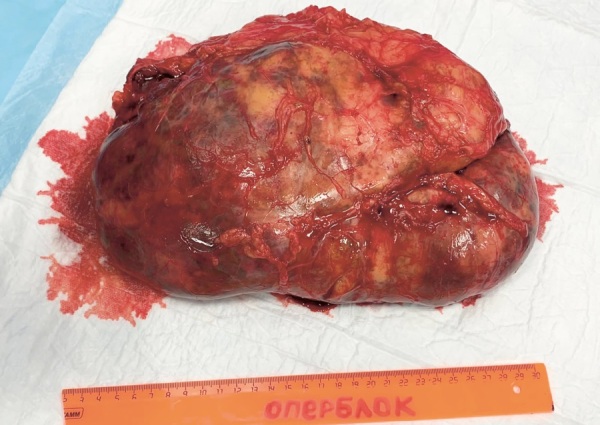
Рисунок 2. Макропрепарат гигантской миелолипомы левого надпочечника при ВДКН.

## Клинический случай №2

Пациент Ч., 35 лет (31.01.1989 г.), в декабре 2023 г. находился на стационарном лечении в ФГБУ «НМИЦ эндокринологии» Минздрава России. На момент госпитализации пациента беспокоили тянущие боли в правом подреберье.

С рождения наблюдался по поводу вирильной формы ВДКН. Диагноз подтвержден результатами молекулярно-генетического анализа. Длительно находится на постоянной заместительной терапии преднизолоном 10 мг/сут. Клинические данные за декомпенсацию отсутствовали.

## Данные физикального обследования

Объективно: состояние удовлетворительное. Масса тела — 74,0 кг. Рост — 152 см. ИМТ — 32,0 кг/кв² (ожирение I). АД — 130/90 мм рт.ст. Пульс — 85 уд./мин. При пальпации в мошонке определяются образования обоих яичек «каменистой» плотности до 1 см. По органам и системам — без особенностей.

Учитывая полученные результаты анализов (табл. 2), свидетельствующие о декомпенсации заболевания: повышение уровней 17OHP и АКТГ, в стационаре проведена коррекция терапии: отмена преднизолона с переводом на дексаметазон 1,25 мг/сут. На фоне скорректированной терапии наблюдалось снижение уровней показателей 17OHP и АКТГ в динамике.

**Table:** Таблица 2. Данные лабораторного обследования пациента №2

Показатель	Результат	Референсный интервал	Единицы измерения
Биохимический анализ крови
Натрий	140,7	136–145	ммоль/л
Хлориды	105,2	98–107	ммоль/л
Калий	3,9	3,5–5,1	ммоль/л
Глюкоза	4,63	3,1–6,1	ммоль/л
Гормональный анализ крови
Тестостерон	12,7	11–28,2	нмоль/л
Андростендион	35,0>	2,1–12,9	нмоль/л
Ренин (прямой)	121>	2,8–39,9	мЕд/л
17-OH прогестерон	669>	0,6–11,8	нмоль/л
АКТГ (утро)	910,9>	7,2–63,3	пг/мл

## Данные инструментального обследования

В связи с жалобами на тянущие боли в правом подреберье выполнена МСКТ забрюшинного пространства: оба надпочечника деформированы, неравномерно утолщены за счет разнокалиберных узелков в структуре размерами до 5–20 мм, неравномерно накапливают контрастный препарат; в медиальной ножке правого надпочечника обнаружено образование с бугристыми четкими контурами размерами до 50х42х42, неоднородное по структуре, с наличием кальцинатов, плотностью 26-86-157-93 HU (нативная — артериальная — венозная — отсроченная фазы соответственно), абсолютный коэффициент вымывания — 48,9%, относительный коэффициент вымывания — 40,8%, в левом надпочечнике — аналогичное образование размерами 22х25х18 мм (рис. 3).

**Figure:**
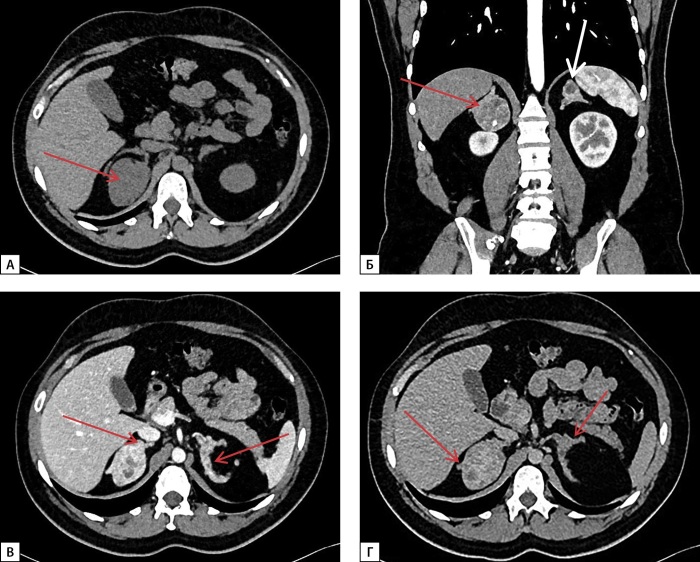
Рисунок 3. МСКТ надпочечников с контрастным усилением: А — нативная фаза — в медиальной ножке правого надпочечника образование с бугристыми четкими контурами, неоднородное по структуре, с наличием кальцинатов (красная стрелка); Б — артериальная фаза — в левом надпочечнике аналогичное образование (белая стрелка); В — венозная фаза; Г — отсроченная фаза — оба надпочечника деформированы, неравномерно утолщены за счет разнокалиберных узелков в структуре (красные стрелки).

С учетом злокачественного КТ-фенотипа принято решение о проведении дополнительного обследования — позитронно-эмиссионной томографии с 18F-фтордезоксиглюкозой, совмещенной с КТ (ПЭТ-КТ с ФДГ) для установления злокачественного потенциала образований надпочечников. По данным ПЭТ-КТ с ФДГ от 08.02.2024 г., в правом надпочечнике определялось образование с SUV — 2,6–4,6, в левом — с SUV — 2,39. Проконсультирован эндокринным хирургом, на основании проведенного обследования показаний к хирургическому лечению не было выявлено, рекомендовано динамическое наблюдение.

По данным УЗИ органов мошонки, яички определялись нормальных размеров, эхогенности, придатки не изменены, в области средостения с обеих сторон определялся неоднородной структуры участок справа — 1,5х1 см, слева — 0,7х1 см, что соответствовало двусторонним TART-опухолям. Рекомендовано динамическое наблюдение.

По данным денситометрии (DEXA), минеральная плотность костной ткани (МПК) соответствовала ожидаемым по возрасту значениям (-0,5 SD (по Z-критерию)). Выявлены метаболические осложнения ожирения в виде дислипидемии, неалкогольной жировой болезни печени (НАЖБП).

При контрольном обследовании через 6 месяцев на фоне скорректированной терапии пациент отмечал прибавку массы тела на 4 кг (табл. 3).

**Table:** Таблица 3. Данные лабораторного обследования пациента №2 через 6 месяцев после коррекции терапии

Показатель	Результат	Референсный интервал	Единицы измерения
Биохимический анализ крови
Натрий	138,1	136–145	ммоль/л
Хлориды	99,1	98–107	ммоль/л
Калий	4,55	3,5–5,1	ммоль/л
Гормональный анализ крови
Тестостерон	12,7	11–28,2	нмоль/л
Андростендион	0,28	0,44–4,56	нг/мл
Общий бета-ХГЧ	<1,2	0–5	мМЕ/мл
Альфафетопротеин	0,72	0–5,8	МЕ/м
Ренин (прямой)	290,1>	2,8–39,9	мЕд/л
17-OH прогестерон	8,38	0,6–11,8	нмоль/л
АКТГ (утро)	7,2	7,2–63,3	пг/мл
ЛГ	6,9	2,5–11,0	Ед/л

По данным МСКТ надпочечников от 25.06.2024 г., надпочечники неравномерно утолщены за счет узелков плотностью от -20 ед. Н до 20 ед. Н, сливающихся в конгломераты максимальным размером справа 44х38х32 мм (с кальцинатами и жировыми включениями), слева — 22х15х18 мм. При сравнении с исследованием от 16.10.2023 г. отмечалось уменьшение размеров конгломератов узлов и толщины надпочечников (рис. 4).

**Figure:**
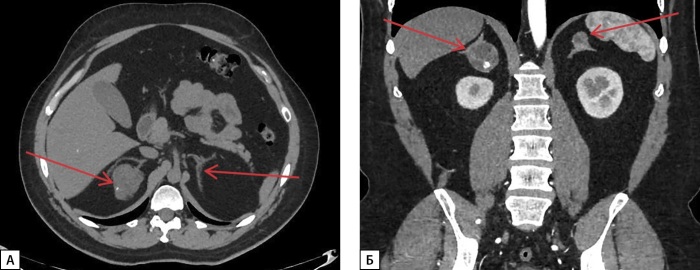
Рисунок 4. МСКТ надпочечников с контрастным усилением: А — нативная фаза, Б — артериальная фаза — отмечается уменьшение размеров конгломератов узлов, уменьшение толщины надпочечников (красные стрелки).

По результатам анализов скорректирована терапия: отменен дексаметазон с переводом на преднизолон 2,5 мг утром и вечером.

Пациент отправлен под динамическое наблюдение.

## Клинический случай №3

Пациентка С., 37 лет (02.01.1987 г.), ежегодно наблюдается в ФГБУ «НМИЦ эндокринологии» Минздрава России по поводу сольтеряющей формы ВДКН.

Диагноз установлен в восьмимесячном возрасте. В возрасте 8 мес. в ЭНЦ выставлен диагноз «ВДКН», назначена терапия преднизолоном в дозе 1,25 мг и дезоксикортикостерона ацетатом (ДОКСА) 0,6–1,0 мг через 2 дня; с 1,5 года переведена на Кортинефф. В 7 месяцев, 2 и 3 года у пациентки отмечались сольтеряющие кризы. В 3 года 9 месяцев проведен 1-й этап феминизирующей пластики. В 9-10 лет отмечались признаки передозировки ГКС — головные боли, стрии, прибавка в весе, а также признаки преждевременного полового развития без наступления менархе. В 15 лет в связи с невозможностью достижения компенсации переведена на Кортеф 30 мг в сут. В 17 лет проведена интроитопластика.

На протяжении всей жизни проводилась коррекция доз ГКС в попытке адаптировать стандартные схемы использования, однако течение заболевания проходило с периодами передозировки и недостаточности доз медикаментозной терапии.

## Данные физикального обследования

Общее состояние удовлетворительное. Телосложение гиперстеническое. Вес — 104, кг. Рост — 154, см. Индекс массы тела — 43,9 (ожирение III ст.). Кожа сухая, отмечается ее мраморность, а также выраженная кожно-жировая деформация в области живота, короткая шея, скошенные ягодицы. Сердечно-сосудистая система: пульс — 90 уд./мин. Артериальное давление — 145/87 мм рт.ст. По остальным органам и системам — без особенностей.

Динамика показателей лабораторного обследования, а также получаемая терапия представлены в таблице 4.

**Table:** Таблица 4. Данные лабораторного обследования и медикаментозной терапии пациентки №3 за период с 2016 по 2023 гг.

Год	Электролиты	Гормональное обследование	Принимаемая медикаментозная терапия
2015	Натрий — 140 ммоль/л, хлориды — 104 ммоль/л, калий — 4 ммоль/л	АКТГ (утро) — 6,5 пг/мл, ренин (прямой) — 9,4 мЕд/л, 17-OHР — 4,6 нмоль/л	Преднизолон 5 мг утром и 3,75 мг вечером Флудрокортизон 0,1 мг 3/4 табл. 2 р/сут
2017	Натрий — 142 ммоль/л, хлориды — 104 ммоль/л, калий — 3,8 ммоль/л	АКТГ (утро) — 10,5 пг/мл, ренин (прямой) — 28,5 мЕд/л, 17-OHР — 21,8 нмоль/л	Преднизолон 5 мг утром и 3,125 мг вечером Флудрокортизон 0,1 мг 3/4 табл. 2 р/сут
2019	Натрий — 139 ммоль/л, хлориды — 108 ммоль/л, калий — 4,4 ммоль/л	АКТГ (утро) — 22 пг/мл, Ренин (прямой) — 70,5> мЕд/л, 17-OHР — 20,1 нмоль/л	Преднизолон 5 мг утром и 3,75 мг вечером Флудрокортизон 0,1 мг 3/4+1/8 табл. утром, 1/4 т вечером
2020	Натрий — 141 ммоль/л, хлориды — 107 ммоль/л, калий — 4 ммоль/л	АКТГ (утро) — 322,4> пг/мл, ренин (прямой) — 71,7> мЕд/л, 17-OHР — 200> нмоль/л	Гидрокортизон по схеме 15-10-5 мг Флудрокортизон 0,1 мг 2 р/сут
2023	Натрий — 138,1 ммоль/л, хлориды — 104 ммоль/л, калий — 3,44< ммоль/л	АКТГ (утро) — 58,36 пг/мл, ренин (прямой) — 17,13 мЕд/л, 17-OHР — 130,8> нмоль/л	Гидрокортизон по схеме 15-10-7,5 мг Флудрокортизон 0,1 мг 2 р/сут

## Данные инструментального обследования

В 2005 г. впервые, по данным КТ надпочечников, диагностировали объемное образование размером 0,8–1,0 см слева. В последующем неоднократно выполняли КТ надпочечников для динамической оценки. На момент последней госпитализации в 2023 г., по данным МСКТ в динамике, в дистальных отделах латеральной и медиальной ножек правого надпочечника определялось образование размерами 25х19х20 мм округлой формы с ровными четкими контурами, наличием кальцинатов в структуре, неоднородной плотности от -4HU до 20HU в нативную фазу исследования, в теле левого надпочечника сохранялось образование жировой плотности (-108HU), с ровными четкими контурами, размерами до 17х12х16 мм. Данные образования трактованы как двусторонние миелолипомы небольшого размера (рис. 5).

**Figure:**
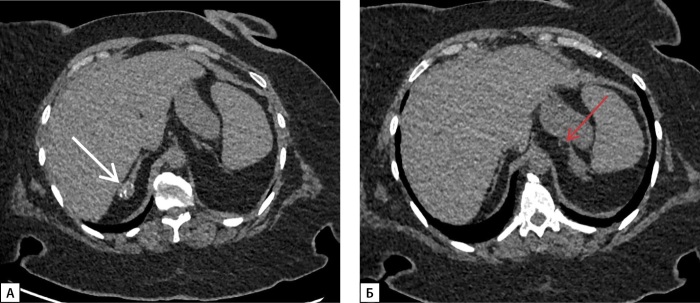
Рисунок 5. МСКТ надпочечников, нативная фаза: А — в дистальных отделах латеральной и медиальной ножек правого надпочечника образование округлой формы с ровными четкими контурами, наличием кальцинатов в структуре (белая стрелка); Б — в теле левого надпочечника образование жировой плотности, с ровными четкими контурами (красная стрелка).

С целью выявления патологии яичников пациентке проведено УЗИ органов малого таза, по данным которого в правом яичнике выявлено анэхогенное образование размерами 4,2х3,7 см (фолликулярная киста?). Со слов, нарушения менструального цикла отсутствовали. Осмотрена гинекологом, рекомендовано динамическое обследование через 3 месяца.

При обследовании у пациентки выявлен комплекс метаболических нарушений: ожирение, дислипидемия, гиперурикемия, НАЖБП и инсулинорезистентность, в связи с чем ежедневно принимала метформин и аллопуринол. По данным DEXA снижения МПК не выявлено.

С учетом доброкачественного характера, а также небольших размеров образований рекомендовано динамическое наблюдение с проведением МСКТ без контрастного усиления через 2 года. Также с учетом наличия у пациентки множественных осложнений не только самого заболевания, но и длительного лечения ГКС, наибольший риск представляла непосредственно сердечно-сосудистая система. В связи с этим рекомендован регулярный мониторинг состояния сердечно-сосудистой системы, включающий контроль артериального давления, липидного профиля, уровня глюкозы, а также своевременную коррекцию выявленных нарушений.

## Клинический случай №4

Пациент Б., 72 года (05.09.1946 г.), в апреле 2019 г. обратился к эндокринному хирургу в ФГБУ НМИЦ эндокринологии с жалобами на боль в поясничной области.

В 1964 г. в возрасте 18 лет поставлен диагноз вирильной формы «ВДКН». Эндокринологом инициирована терапия ГКС (дексаметазон), которую пациент принимал и самостоятельно отменил через 2 года. Со слов больного, в дошкольном возрасте опережал в росте сверстников, затем с 7 лет отмечалось отставание. В период с 20 до 50 лет терапию не получал. В возрасте 50 лет стала беспокоить слабость, быстрая утомляемость и выпадение волос. Не обследовался, не лечился. С 2018 г. стала нарастать выраженная слабость, появились боли в пояснице. В связи с чем обратился за медицинской помощью, после дообследования (медицинская документация не предоставлена) эндокринологом возобновлена заместительная терапия (преднизолон 5 мг утром, 2,5 мг вечером).

## Данные физикального обследования

При осмотре обращает внимание низкорослость — рост составляет 145 см. Вес — 62,0 кг (ИМТ — 29). Пульс — 72 уд./мин. Артериальное давление — 145/87 мм рт.ст. По остальным органам и системам — без особенностей.

## Данные лабораторного обследования (табл. 5) (на фоне терапии преднизолоном 7,5 мг/сут)

По результатам представленных данных, с учетом клиническом картины, доза ГКС терапии адекватна. Выявлены возрастные признаки первичного гипогонадизма (повышение ЛГ, ФСГ, снижение тестостерона), коррекция которых не требовалась.

**Table:** Таблица 5. Данные лабораторного обследования пациента №4 при первичном обращении (апрель 2019 г.) через 6 месяцев после коррекции терапии

Показатель	Результат	Референсный интервал	Единицы измерения
Биохимический анализ крови
Натрий	141	136–145	ммоль/л
Хлориды	103	98–107	ммоль/л
Калий	4,8	3,5–5,1	ммоль/л
Гормональный анализ крови
Тестостерон	0,42	2,4– 6,7	мкг/л
Андростендион	1,05	0,44–4,56	нг/мл
17-OH прогестерон	7,45	0,6–11,8	нмоль/л
АКТГ (утро)	77	7,2–63,3	пг/мл
ЛГ	51	2,5 –11,0	Ед/л

## Данные инструментального обследования

Также в апреле 2019 г. при МСКТ впервые выявлено образование правого надпочечника размерами 11х12х12 см с четкими ровными контурами, без признаков инвазивного роста, неоднородной структуры, плотностью от 5 до 40 ед.Н, накопление только периферическими участками опухоли, в области левого надпочечника — образование размерами 2,2х1,7х1,6 см, с нативной плотностью 13 HU (рис. 6).

**Figure:**
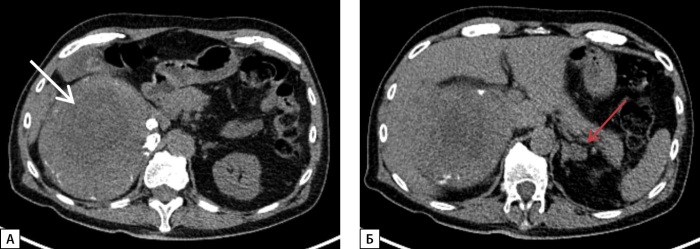
Рисунок 6. МСКТ надпочечников, нативная фаза: А — образование правого надпочечника округлой формы с ровными четкими контурами, наличием кальцинатов в структуре (белая стрелка); Б — образование левого надпочечника с ровными четкими контурами (красная стрелка).

С учетом размера образования, а также наличия высокоплотных участков рекомендовано проведение ПЭТ-КТ с ФДГ, которое пациенту выполнено в мае 2019 г. По данным вышеуказанного исследования, отмечалось неравномерное накопление SUV 2,48–2,59–3,93 по периферии тканевым компонентом правого надпочечника; образованием латеральной ножки левого надпочечника 1,7х1,3 см — SUV 3,27; гипофизом (без наличия образования) — SUV 5,16.

Принято решение о динамическом наблюдении пациента с повторной оценкой размера образований надпочечника через 6 месяцев.

При контрольной явке пациент отмечал нарастание болей в поясничной области. При МСКТ надпочечников без контрастного усиления в октябре 2019 г. в области правого надпочечника выявлено объемное образование неоднородной структуры, плотностью от 16 до 46 HU, размерами 11,9x13,2x12,5 см, в структуре образования визуализировались множественные кальцинаты, преимущественно на периферии, без признаков инвазивного роста, в латеральной ножке левого надпочечника — образование с ровными четкими контурами размерами 2,0x1,7x1,8 см, плотностью от 16 до 23 HU.

С учетом усиления болей в поясничной области, после исключения других причин болевого синдрома, принято решение об оперативном лечении пациента.

02.12.2019 г. под общим обезболиванием выполнена правосторонняя торакофренолапаротомия, правосторонняя адреналэктомия с опухолью.

Послеоперационный период протекал без осложнений. Пациент выписан в удовлетворительном состоянии на 5-е сутки после оперативного вмешательства.

При макроскопическом исследовании послеоперационного материала на разрезе отмечалась опухоль с жировой клетчаткой размером 15,0 см в наибольшем измерении (макропрепарат представлен на рис. 7), режущаяся с хрустом. По данным микроскопического исследования, к истонченному надпочечнику прилежала четко отграниченная тотально некротизированная опухоль без признаков васкулярной и капсулярной инвазии, местами инфильтрированная лейкоцитами, с очагами кровоизлияний и оссификации по периферии опухоли. Морфологическая картина соответствовала тотально некротизированной опухоли надпочечника.

**Figure:**
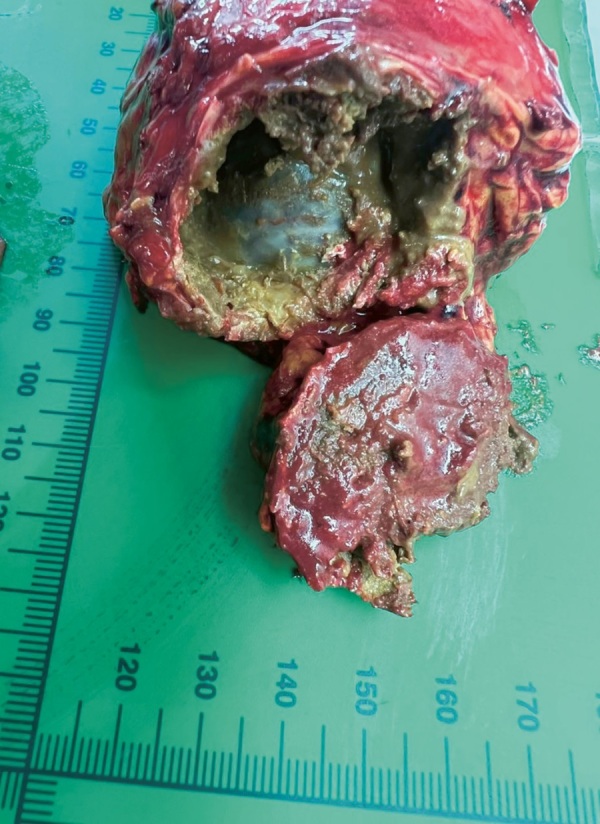
Рисунок 7. Макропрепарат: тотально некротизированная опухоль правого надпочечника пациента с ВДКН.

## ОБСУЖДЕНИЕ И ЗАКЛЮЧЕНИЕ

ВДКН характеризуется сложными гормональными нарушениями, потенциально опасными для жизни и приводящими к серьезным осложнениям. Несмотря на развитие заместительной терапии, контроль заболевания остается сложной задачей, что подтверждают представленные клинические случаи.

Распространенность образований надпочечников среди пациентов с ВДКН достаточно велика и достигает, по данным Reisch и соавт. [9], 82%. В представленном Nermoen и соавт. метаанализе у 29,3 % лиц с ВДКН выявлены опухоли надпочечников, четверть из которых были представлены миелолипомами [7]. Установлено, что увеличение общего объема надпочечников и их гиперплазия коррелируют с неудовлетворительным контролем и неблагоприятными долгосрочными исходами [8][9][10]. Как было упомянуто ранее, высокий уровень АКТГ действует как фактор роста на клетки надпочечников и, таким образом, приводит к гиперплазии надпочечников [8][10][12]. Является ли повышенный уровень АКТГ также причиной формирования роста опухолей, на данный момент неясно, но это может быть одной из причин высокой распространенности миелолипом и аденом надпочечников при ВДКН [9]. Было обнаружено, что АКТГ может способствовать, по крайней мере, развитию и росту миелолипомы [12] (рис. 8).

**Figure:**
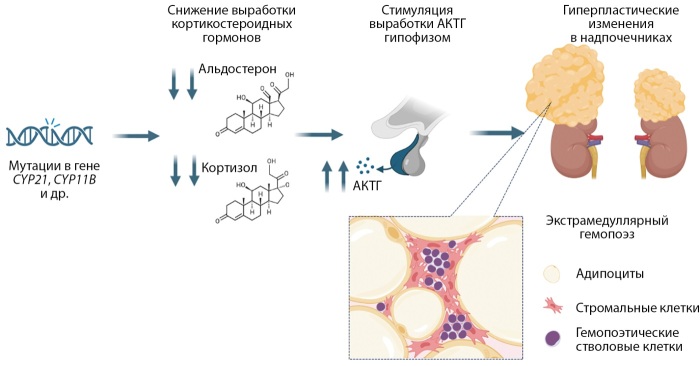
Рисунок 8. Потенциальный патогенетический механизм развития миелолипом надпочечников при ВДКН. ВДКН развивается в результате генетической мутации, чаще всего затрагивающей ген, кодирующий фермент 21-α-гидроксилазу, что приводит к нарушению стероидогенеза и недостаточной выработке двух ключевых кортикостероидов — кортизола и альдостерона. В условиях данного дефицита гипофиз, по механизму обратной отрицательной связи, усиливает продукцию АКТГ. Длительное повышение уровня АКТГ может индуцировать экстрамедуллярный гемопоэз в надпочечниках, способствуя замещению адренокортикальной ткани миелоидной, что, в свою очередь, приводит к развитию и прогрессирующему росту миелолипом. Изображение создано в программе Biorender.com.

Миелолипома надпочечников — относительно редкое доброкачественное образование, содержащее в основном жир и кроветворные элементы, напоминающие костный мозг. При КТ для миелолипом характерна мозаичная структура: показатели нативной плотности макроскопических участков жира имеют отрицательные денситометрические показатели (<0 HU), в свою очередь для миелоидного компонента характерна высокая плотность (>15HU). В некоторых случаях миелолипомы практически полностью представлены миелоидным компонентом, что затрудняет их дифференциальную диагностику с аденомами и адренокортикальным раком (АКР). Хотя АКР при ВДКН встречается крайне редко, и в настоящее время нет данных об увеличении риска развития данного состояния у пациентов с ВДКН, тем не менее описаны случаи сочетания миелолипом и АКР [13][14][15].

В большинстве случаев ПЭТ-КТ с ФДГ позволяет провести дифференциальную диагностику между различными образованиями надпочечников с неопределенным и злокачественным КТ-фенотипом. Высокая метаболическая активность (SUV более 3,5 или превышающая SUV печени в 1,5 раза) служит критерием, с высокой вероятностью указывающим на злокачественный характер опухоли. При ПЭТ-КТ миелолипомы обычно демонстрируют поглощение ФДГ ниже, чем в печени [16][17]. В редких случаях миелолипома с обширными аденоматозными и кроветворными элементами может демонстрировать высокое поглощение ФДГ [18][19]. В клинических наблюдениях №2, 4, учитывая размеры и высокую плотность образований, с целью дифференциальной диагностики выполнено ПЭТ-КТ, что позволило установить низкий злокачественный потенциал образований. При наличии у пациента с ВДКН повышенного накопления образованием надпочечника ФДГ при ПЭТ-КТ в качестве дополнительного диагностического критерия может быть использована оценка динамики роста образования по КТ. Миелолипомы и аденомы отличаются относительно медленным ростом в отличие от АКР. В большинстве случаев больным с объемными образованиями надпочечника при ВДКН не требуется хирургическое лечение. Основным показанием к оперативному лечению является синдром механической компрессии органов брюшной полости, что представлено в клинических случаях №1, 4. Другими показаниями могут быть некомпенсируемая гиперандрогенемия у женщин и — в редких случаях —невозможность дифференциальной диагностики со злокачественным образованием.

Длительная декомпенсация заболевания, ассоциированная с недостаточной дозой ГКС, может приводить к развитию TART-опухолей — очагов адренокортикальной ткани в яичках. Онтогенетически TART рассматриваются как аберрантные адренокортикальные клетки, которые мигрируют вместе с гонадами из мочеполового гребня во время развития плода; однако они также могут происходить из плюрипотентных стероидогенных клеток или быть предшественниками клеток Лейдига [1]. TART в основном двусторонние, и в среднем встречаются у 37% мужчин с классической врожденной гиперплазией надпочечников. Распространенность TART увеличивается с возрастом и связана с типом мутации CYP21A2. Дифференциальная диагностика между TART и опухолями клеток Лейдига затруднена [20]. Динамическое наблюдение пациентов сводится в первую очередь к оценке тестикулярной функции и роста опухолей по данным УЗИ или МРТ мошонки. Рост образований, как правило, приводит к сдавливанию сети яичка, перитубулярному фиброзу, гиалинизации, а также лимфоцитарной инфильтрации и, как следствие, необратимому повреждению паренхимы тестикул. В представленных клинических случаях №1 и №2 у обоих пациентов выявлены TART-опухоли, однако у пациента, длительно находившегося в состоянии декомпенсации, образования яичек были значительно больше, в связи с чем можно предполагать некую корреляцию между уровнями АКТГ, длительностью декомпенсации и размерами опухолей. По данным Stikkelbroeck и соавт., на фоне интенсификации терапии ГКС можно ожидать регресс TART, особенно если они обнаружены на ранней стадии [21][22].

Представленные наблюдения подчеркивают необходимость тщательного мониторирования пациентов с ВДКН, своевременной коррекции доз заместительной терапии и использования современных методов визуализации для оценки надпочечниковых образований. Ключевыми аспектами улучшения прогноза и качества жизни пациентов с ВДКН являются оптимизация терапевтических схем, обеспечение эффективного перехода из педиатрической службы под наблюдение врачей-эндокринологов для взрослых, а также длительное психологическое сопровождение на всех этапах жизни. При этом последнее не только способствует улучшению психоэмоционального состояния, но и играет важную роль в повышении приверженности пациентов к лечению, что существенно влияет на снижение риска долгосрочных осложнений. Необходимы дальнейшие исследования для разработки более точных терапевтических стратегий, учитывающих особенности гормональной регуляции у пациентов с ВДКН.

## ДОПОЛНИТЕЛЬНАЯ ИНФОРМАЦИЯ

Источники финансирования. Работа выполнена в рамках государственного задания «Разработка новых технологий диагностики и мониторинга опухолей коры надпочечников с использованием метаболомных и протеомных технологий». Регистрационный номер 123021300098-7.

Конфликт интересов. Авторы декларируют отсутствие явных и потенциальных конфликтов интересов, связанных с содержанием настоящей статьи.

Участие авторов. Все авторы одобрили финальную версию статьи перед публикацией, выразили согласие нести ответственность за все аспекты работы, подразумевающую надлежащее изучение и решение вопросов, связанных с точностью или добросовестностью любой части работы.

Согласие пациента. Пациенты добровольно подписали информированное согласие на публикацию персональной медицинской информации в обезличенной форме.
